# Violence in the Emergency Department: What Can We Do?

**DOI:** 10.7759/cureus.41909

**Published:** 2023-07-14

**Authors:** Ayse Dilara Oztermeli, Ahmet Oztermeli, Emre Şancı, Hüseyin Cahit Halhallı

**Affiliations:** 1 Emergency Medicine, Gebze Fatih State Hospital, İzmit, TUR; 2 Orthopedics and Traumatology, Gebze Fatih State Hospital, İzmit, TUR; 3 Emergency Medicine, Kocaeli City Hospital, İzmit, TUR

**Keywords:** health system development, security, health worker, emergency department, violence

## Abstract

Background: Violence in healthcare settings is a problem around the world, with hospital emergency departments (EDs) being the most common sites. The most important step in preventing violence is to determine the causes and characteristics of the problem. However, there is not enough information in the literature about the particular areas of EDs in which violence occurs.

Objectives: We aim to produce results that can contribute to violence prevention activities by gathering detailed information about violent incidents in EDs and the intensity of this violence.

Methods: Our study was planned as a retrospective and descriptive study at a tertiary emergency medicine clinic. Our data include “code white” data between January 1, 2015, and December 31, 2019. The characteristics and types of violence were recorded and categorized.

Results: We evaluated 141 incidences of severe violence reported during the study period. We determined that 55.2% of the violence was directed at physicians and 21.3% at nurses. Verbal violence was by far the most common type of violence, comprising 98.6% of the cases. We found that the violence cases occurred in examination rooms, observation areas, and triage units of the ED (58.2%, 24.1%, and 11.3%, respectively).

Conclusion: We determined that violence in the ED is most common after standard working hours, and the most frequent exposure to violence is in examination rooms, observation areas, and triage areas. These findings may be useful in determining preventive measures in EDs, where violence is most common.

## Introduction

The bond of trust between the patient and the physician is one of the conditions that form the basis of medical practices dating back to ancient times. However, violence in healthcare settings has increased in recent years and has affected this trusted bond. The healthy relationship between the patient and the physician deteriorates with the weakening of the trust bond [1].

Violence against healthcare workers is most common in developing countries such as India, China, Pakistan, and Nepal [2-4]. It has been demonstrated that doctors in India are exposed to violence at a rate as high as 75% [5]. Violence in the health field is also an important problem in developed countries. A study conducted in the United States found that healthcare workers were exposed to violence at a higher rate than members of other working groups [6]. According to a World Health Organization report, healthcare workers are exposed to physical violence for between 8% and 38% of their working lives [5].

In the country in which our study was conducted, a study carried out in emergency departments in 2002 reports verbal violence rates that can reach 61.1% within the last five years [7]. Emergency departments (EDs) are the riskiest areas in terms of exposure to violence [8,9]. In a study investigating violence against healthcare workers, ED workers were exposed most frequently to violence, at a rate of 64.2% [10]. However, no study examining violence against healthcare workers has discussed the intensity of violence among ED departments specifically.

We believe it is important to determine the type of violence-psychological, physical, verbal, and sexual-and the location of its occurrence in ED. EDs are organic structures consisting of many parts. The analysis of violent events through the data recorded in the ED structure can contribute to the healthier functioning of this organic structure. Our aim in this study is to examine the reports of violence over a certain period in a tertiary ED to analyze the causes of violence against healthcare workers and to produce results that can contribute to violence prevention activities.

## Materials and methods

Our study was planned as a retrospective and descriptive study at a tertiary academic emergency medicine clinic. Local ethics committee approval was received for this study (Approval No: 2019-142). All reports of violence that occurred between January 1, 2015, and December 31, 2019, were examined. Violence in the healthcare field begins with activating the “code white” system within the framework of local regulations. "Code white" is a protocol in our country where crimes committed against healthcare personnel during the provision of healthcare services, as well as related proceedings and lawsuits, are recorded. For this reason, code white data is used in our study.

To systematize the reports of violence, the following data about violent incidents were analyzed:

1. The date and time of the violence;

2. The place where the violence occurred (ED area);

3. The type of violence and mechanism, if any;

4. The text regarding the violence in the report;

5. The title of the healthcare worker exposed to the violence;

6. The age and gender of the healthcare worker exposed to the violence;

7. The seniority of the healthcare worker exposed to the violence;

8. The gender of the healthcare worker exposed to the violence; and the gender of the perpetrator, such as patient, patient’s relative, or other.

The types of violence are classified as follows:

1. Psychological violence: intentional pressure, including intimidation (threat), by making a person or group feel as if physical force could be used that could harm their physical, mental, spiritual, moral, or social development;

2. Verbal violence: the use of words and gestures to intimidate, suppress, and punish;

3. Physical violence: the presence of lesions and injuries that adversely affect the health of the victim and leave marks on the body from abuse that starts with beating using physical force; and

4. Sexual violence: an offensive behavior that causes fear, humiliation, and embarrassment to the person concerned, as well as unwanted, unanswered, and unwelcome physical, psychological, or verbal expression, written and visual materials, and all types of sexual expression.

The causes of violence are categorized as follows:

1. Not wanting to wait in line

2. Not requesting/approving the necessary procedures for diagnosis

3. Not wanting/not confirming the treatment

4. Not wanting to leave the treatment area

5. Gender problem

6. Improper requests

7. Problems arising from technical deficiencies in the hospital

8. Problems with counseling

9. Other

Statistical analysis

The data were analyzed statistically using the IBM SPSS 20.0 (IBM Corp., Armonk, NY, USA) package program. The normal distribution of the parameters was evaluated by the Shapiro-Wilk test. The numerical variable was given a mean ± standard deviation, and the categorical variable was given a frequency. Differences between groups for the evaluation of categorical variables were evaluated with Pearson and Monte Carlo chi-square tests. Significance was assessed at p <0.05.

## Results

We evaluated 141 reports of violence recorded during the study period.

When the reports of violence against healthcare workers were examined, it was determined that physicians were affected the most, as they were targeted in 55.2% of the incidents. Among physicians, the medical residents were exposed to the highest frequency of violence, with 73.7% of the reports. Nurses were the target of 21.3% of violent incidents.

As for the gender of the physicians exposed to violence, 50.6% were male and 49.4% were female. The age of the victims ranged from 20 to 60 years old, with the average age being 32.1.

When the seasons were analyzed, the highest incidence of violence occurred in the summer, with 31.9%, and the lowest in the spring, with 20.6%. The violence took place most commonly between 16:00 and midnight and was most frequently seen in the examination rooms, at a rate of 58.2%, followed by observation areas at 24.1%, and triage units at 11.3% (Table 1).

**Table 1:** Evaluation of code white data according to season, time of incident, and place of violence.

	n = 141	%
Season		
Winter	36	25.5
Spring	29	20.6
Summer	45	31.9
Autumn	31	22.0
Time of incident		
08:00–16:00	39	27.9
16:00–00:00	78	55.7
00:00–08:00	23	16.4
Area		
Triage	16	11.3
Examination rooms	82	58.2
Observation areas	34	24.1
Other	9	6.4

When the types of violence were examined, verbal violence was found in 98.6% of the cases, psychological violence in 51.8%, and physical violence in 19.1%. No cases of sexual violence were reported. When the cases of physical violence are examined, hitting and pushing are the most common, at 55.6%, followed by throwing materials, at 33.3% (Table 2).

**Table 2:** Evaluation of code white data by type of violence.

	n = 141	%
Verbal violence	139	98.6
Physical violence	27	19.1
Hitting, squeezing	15	55.6
Throwing material	9	33.3
Pushing	0	0
Unspecified	3	11.1
Psychological	73	51.8

When the reasons leading to violence were examined, the highest rate was associated with not wanting to wait in line for an examination (36.9%), followed by cases of not wanting/not agreeing to treatment (9.9%). Nearly one-third of patients who objected to waiting in line for their examination (30.8%) resulted from requests to turn the green triage applied to the patients to yellow (Table 3).

**Table 3:** Evaluation of code white data according to the causes of violence.

	n = 141	%
Not wanting to wait in line	52	36.9
In triage	16	30.8
Not in triage	36	69.2
Not wanting/not liking the procedures required for diagnosis	13	9.2
Not wanting/disliking the treatment	14	9.9
Not wanting to leave the treatment area	6	4.3
The sexist problem	2	1.4
Best request	3	2.1
Problems caused by the lack of technical facilities in the hospital	7	5.0
Problems caused by consultation	6	4.3
Other	38	27.0
Total	141	100.0

When the seniority of the healthcare worker was compared, the highest frequency of violence was directed at physicians with 0-4 years of seniority (p < 0.001) (Table 4).

**Table 4:** Evaluation of code white data according to the title and seniority of the healthcare worker who has been subjected to violence.

			Physician	Nurse	Other healthcare workers	Other	Total
Seniority							
	0–4 Years	n	38	5	0	3	46
		%	82.6%	10.9%	0.0%	6.5%	100.0%
	5–10 Years	n	42	20	14	8	84
		%	50.0%	23.8%	16.7%	9.5%	100.0%
	Over 10 years	n	16	12	13	3	44
		%	36.4%	27.3%	29.5%	6.8%	100.0%
	Total	n	96	37	27	14	174
		%	55.2%	21.3%	15.5%	8.0%	100.0%

As for the perpetrators’ identities, 73.8% are the patients’ relatives and 26.3% are the patients themselves. By gender, 70.4% of the perpetrators are male and 26.4% are female.

## Discussion

Physicians and nurses are the groups most exposed to violence among healthcare workers. In a meta-analysis covering 253 cases, Liu et al. determined that the groups most exposed to violence were nurses with 59.2%, followed by physicians with 56.8%, and other healthcare workers with 44.4% [11]. In their study, which included the analysis of 290 code white data points provided between 2012 and 2015, Avcı et al. detected violence against physicians at 63%, against nurses at 22%, and against other healthcare personnel at 15% [12]. The rates of violence against these groups in our research were similar to those reported in other studies. This can be explained by the fact that physicians and nurses are in close contact with patients and their relatives. When we evaluated the occurrence of violence against physicians, we found the highest rate (73.7%) against medical residents. This is because the hospital at which the study was conducted is a training clinic, and resident physicians handle most patient admissions.

There are quite different results in the literature regarding the gender of victims of violence. In a meta-analysis of 253 cases by Liu et al., no statistically significant difference was found regarding the prevalence of physical or non-physical violence by gender [11]. In a survey study involving 254 physicians and nurses, Kaya et al. found the violence rate to be 76.6% for men and 73.4% for women, and they could not detect a statistical significance between these numbers [13]. In a study conducted by Polat and Çırak of 345 code white cases at a local hospital in Istanbul between 2016 and 2018, it was determined that 63.18% of female professionals and 36.81% of male professionals were exposed to violence [14]. By contrast, our study found nearly equal victimization rates for the two genders: 50.6% for male professionals and 49.4% for female professionals.

The exposure of victims of violence is inversely proportional to age and professional experience. In a survey of 700 professionals from Baghdad, researchers Lafta and Falah found that rates of physical violence are statistically significantly higher among younger people [15]. Our results were similar, with greater rates of violence found among young people with low seniority. This could be because medical professionals with high seniority have better communication skills and a greater ability to cope with stress. Alternatively, it is possible that fewer reports of violence are filed by potentially burned-out professionals who have spent many years on the job. More comprehensive studies need to be conducted to support our views on this. Ayranci et al., who surveyed 1,209 professionals at 34 hospitals in four provinces, determined that violence was the highest among professionals who had been working in the sector for fewer than 10 years; they found this result to be statistically significant [10]. Lafta and Falah also discovered in their study of 700 professionals in Baghdad that violence was higher among professionals with less work experience [15]. By comparing the employees’ levels of seniority, we also determined a statistically significant rate of high violence exposure among employees with 0-4 years of service.

Violence in the healthcare field is seen most frequently in the summer. Avcı et al., in their study of 290 code white cases between 2012 and 2015, found that the most violent cases occurred in the spring and summer [16], but they did not detect a statistically significant difference. In the case studies they obtained from a Chinese database, Teoh and colleagues determined that 47.2% of 235 incidents of violence were committed between May and August, confirming summer as the most violent season [17]. Our study, in which the greatest number of cases were detected in the summer, can be seen as similar to other studies in the literature. Hsiang et al. found that each 1-SD change in climate toward warmer temperatures increased the frequency of interpersonal violence by 4% and the violence between groups by 14% [18]. We believe that heat be the reason for higher rates of violence during the summer. It should not be ignored that seasonal differences may vary according to geographical regions, and this situation may include factors other than temperature change. Nevertheless, it can be concluded that seasonal differences may emerge with more comprehensive studies that consider all factors.

Violent incidents generally occur during standard working hours. In a study conducted by Vural et al. at a local hospital in Kocaeli, Turkey, staffed by 50 physicians, it was determined that the most frequent occurrence of violence was during working hours, between 08:00 and 16:00 [19]. In a study by Polat and Çırak, the period when the number of cases peaked was determined as 08:00-16:59 as same [14]. In our study, violence occurred predominantly between 16:00 and midnight. However, we reached this conclusion by examining the data obtained only from the code white cases originating from ED. We believe the differences in findings may be because the patients applied to the ED after working hours. Except for ED employees, all departments work actively during regular working hours at the hospital. If we had examined the code white data reported in all departments, we might have produced findings similar to those of other studies. However, our result is valuable because it pinpoints the hours when violence intensifies in EDs.

It is known that incidents of in-hospital violence are most common in EDs [10,20]. Because of our study’s focus on violence in the ED, we examined the distribution of violence cases according to particular departments in the ED. Our data revealed that the most violent cases were in examination rooms, observation areas, and triage units (58.2%, 24.1%, and 11.3%, respectively). We believe this information is vital for hospitals to take preventive measures against violence. No previous study has examined violence in specific emergency service areas. In this respect, our unique findings can serve as a guide in determining the underlying causes of violence in EDs and in developing actions to prevent violence. We theorize that violence is most prevalent in examination rooms because these areas are the places where patients spend the most time and where the first information about their medical condition is determined. Violence in the triage areas, which ranked third for frequency, was somewhat surprising. In the health facility where the study was conducted, nurses perform triage. Patients’ dislike of the green, yellow, and red color-coded triage system seems to be the root of the violence in this area.

It should be noted that our study is based on actual case reports. Apart from emergency cases applying to EDs, we believe the growing number of applications due to difficulties accessing other parts of hospitals may also be a cause of violence. When we evaluate the violent incidents stemming from the patients’ unwillingness to wait, 30.8% of these cases occur in triage because they want to change their green area triage to the yellow area. Our study is unique in that it separately assesses ED areas.

Our study evaluated the different types of violence by classifying them. A meta-analysis by Liu et al. determined that non-physical violence against doctors comprised 42.5% of the total number of incident reports, and the most common type was verbal abuse, at 57.6% [11]. In their survey of 597 physicians at seven hospitals, Baykan et al. determined that verbal abuse was the most common type of violence, comprising 96.8% of the total number of incident reports. The next most common type of violence against healthcare personnel is physical violence, with 26.8% [21]. In our study, verbal abuse was found to be the most common (98.6%), and it was evaluated in a way that would be similar to other studies in the corpus. Among the reasons for the prevalence of verbal violence are that verbal acts are easier to perform, the occurrence of verbal acts is less provable than physical acts, people believe the realization of such acts will not constitute a crime, and, therefore, people believe that a perpetrator likely will not be punished. In addition, the fact that such acts against healthcare personnel are not seen as crimes and, therefore, are not subject to any punitive actions leads one to conclude that this type of violence will only worsen.

Patient companions, mostly men, are most frequently responsible for violent incidents [10,21]. In our study, patient companions were found to be the largest group of violent perpetrators, participating in 73.8% of the incidents, with 70.4% of the companions being men. This is a result consistent with the literature. Scholars theorize that patients’ relatives are more volatile because they expect the patients to recover quickly and believe these urgent expectations are not being met.

Study limitations

The limitations of our study include the single-center nature of our study, the evaluation of data obtained only from the ED, and the exclusion of cases not reported within the framework of the code white data.

## Conclusions

In this study, we aimed to produce results that can contribute to violence prevention activities by gathering detailed information about violent incidents in EDs and the intensity of this violence. First, measures to reduce ED overcrowding are needed across the entire health system. It may also be beneficial to provide regular training to increase healthcare professionals’ ability to cope with stressful situations and maintain communication with aggressive people. In addition, posting information about employee rights adjacent to existing information about patient rights and increasing the penalties for violence through new laws can help reduce these incidents. Finally, violence may be reduced by providing a sufficient number of healthcare personnel at every institution, reducing working hours, improving physical conditions, and making patients and their family companions more comfortable.
